# Refractory Chronic Knee Pain After Total Knee Replacement Successfully Treated With L4 Dorsal Root Ganglion Pulsed Radiofrequency

**DOI:** 10.7759/cureus.102951

**Published:** 2026-02-04

**Authors:** Mohamad Omar, Benan M AlShehhi, Salah Al Ali

**Affiliations:** 1 Anesthesiology, Emirates Health Services, Sharjah, ARE; 2 Anesthesiology, Dubai Health, Rashid Hospital, Dubai, ARE; 3 Anesthesiology and Pain Medicine, Dubai Health, Rashid Hospital, Dubai, ARE

**Keywords:** chronic knee pain, chronic lumbar radiculopathy, dorsal root ganglion, genicular nerve, l4 nerve root, neuropathic pain, postoperative pain, pulsed radiofrequency treatment, total knee replacement (tkr)

## Abstract

Persistent pain following total knee replacement (TKR) remains a significant clinical problem and may lead to long-term functional impairment despite technically successful surgery. The underlying mechanisms are often multifactorial and may include neuropathic and referred pain components. We report the case of a 71-year-old woman with severe bilateral knee pain persisting after bilateral TKR, refractory to pharmacologic therapy, physiotherapy, genicular nerve interventions, and peripheral nerve blocks. Ongoing neuropathic features and failure of peripheral treatments raised suspicion of a spinal pain source. A diagnostic left L4 selective nerve root block produced marked but temporary pain relief, supporting a radicular pain component. Subsequent therapeutic pulsed radiofrequency (PRF) of the left L4 dorsal root ganglion (DRG) resulted in sustained pain reduction exceeding 70%, functional improvement, and discontinuation of analgesic medications. This case emphasizes the importance of considering spinal pain generators in refractory post-TKR pain and suggests DRG-targeted PRF as a potential treatment option in appropriately selected patients.

## Introduction

Chronic knee pain, defined as pain persisting for more than three months, is a leading cause of global disability, particularly among older adults [1]. Although knee osteoarthritis is the most common etiology, knee pain may also arise from extra-articular sources, including peripheral neuropathies and referred spinal pathology [2]. Lumbar radiculopathy, especially involving the L3 and L4 nerve roots, is frequently underrecognized due to overlapping dermatomal innervation of the anterior and medial knee [3].

Radicular pain refers to pain originating from irritation or dysfunction of a spinal nerve root and typically follows a dermatomal distribution, whereas referred pain arises from spinal or deep somatic structures but is perceived at a distant site without a clear dermatomal pattern. Differentiating between these entities can be clinically challenging, particularly in the postoperative setting, where symptoms may overlap with peripheral or joint-related pain [4].

Total knee replacement (TKR) is widely considered the definitive treatment for end-stage knee osteoarthritis, with hundreds of thousands of procedures performed annually worldwide [5]. Despite favorable surgical outcomes, persistent postoperative pain affects approximately 10% to 20% of patients [6]. Neuropathic pain features such as burning sensations, electric shocks, and allodynia have been reported in 6% to 13% of post-TKR patients and are associated with poorer functional outcomes and reduced satisfaction [7].

Increasing evidence suggests that sensitization of the dorsal root ganglion (DRG), a key structure involved in sensory signal transmission, may contribute to persistent neuropathic and radicular pain states [8]. Pulsed radiofrequency (PRF) is a non-destructive neuromodulatory technique that delivers intermittent electrical fields to neural tissue, aiming to reduce pain through modulation of neuronal signaling rather than nerve ablation [9].

Management of chronic post-TKR pain is challenging and often requires a multimodal approach. While peripheral interventions such as genicular nerve blocks and radiofrequency ablation are commonly employed, a subset of patients remains refractory, suggesting the involvement of alternative pain generators. Increasing evidence supports the role of DRG sensitization and radicular pain mechanisms in persistent postoperative knee pain [10].

## Case presentation

A 71-year-old woman with a history of essential hypertension and bilateral end-stage knee osteoarthritis underwent bilateral total knee replacement in 2022. The immediate postoperative course was surgically uncomplicated; however, she reported persistent bilateral knee pain beginning shortly after surgery, with gradual worsening over time. She presented to our pain clinic in October 2023 with severe chronic knee pain, more pronounced on the left side. The pain was described as burning, cramping, and shooting in nature, consistent with neuropathic characteristics. Baseline pain intensity was rated as 8/10 on the Numerical Rating Scale (NRS), escalating to 10/10 with prolonged standing, sitting for more than 15 minutes, walking, or side-lying positions. The pain was constant and associated with tingling, dysesthesia, and marked allodynia involving both the medial and lateral aspects of the knees. These symptoms caused significant sleep disturbance, impaired mobility, and limitation of daily activities. Initial conservative management included pharmacologic therapy with celecoxib and duloxetine, both of which were discontinued due to inadequate pain relief and adverse effects. The patient completed multiple courses of physiotherapy, including transcutaneous electrical nerve stimulation, without sustained benefit. In April 2023, she underwent bilateral cooled radiofrequency ablation of the genicular nerves, which resulted in only transient relief lasting approximately four days. Physical examination revealed pronounced cutaneous allodynia and dysesthesia around both knees, more severe on the left side. Motor strength, reflexes, and knee joint range of motion were preserved, with no evidence of instability. The initial diagnosis was chronic postoperative knee pain with a significant neuropathic component, raising concern for saphenous and common peroneal nerve involvement. However, the presence of burning and shooting pain, dermatomal distribution, symptom exacerbation with prolonged sitting and positional changes, and incomplete response to peripheral nerve-targeted interventions raised suspicion for a proximal radicular pain source.

**Figure 1:**
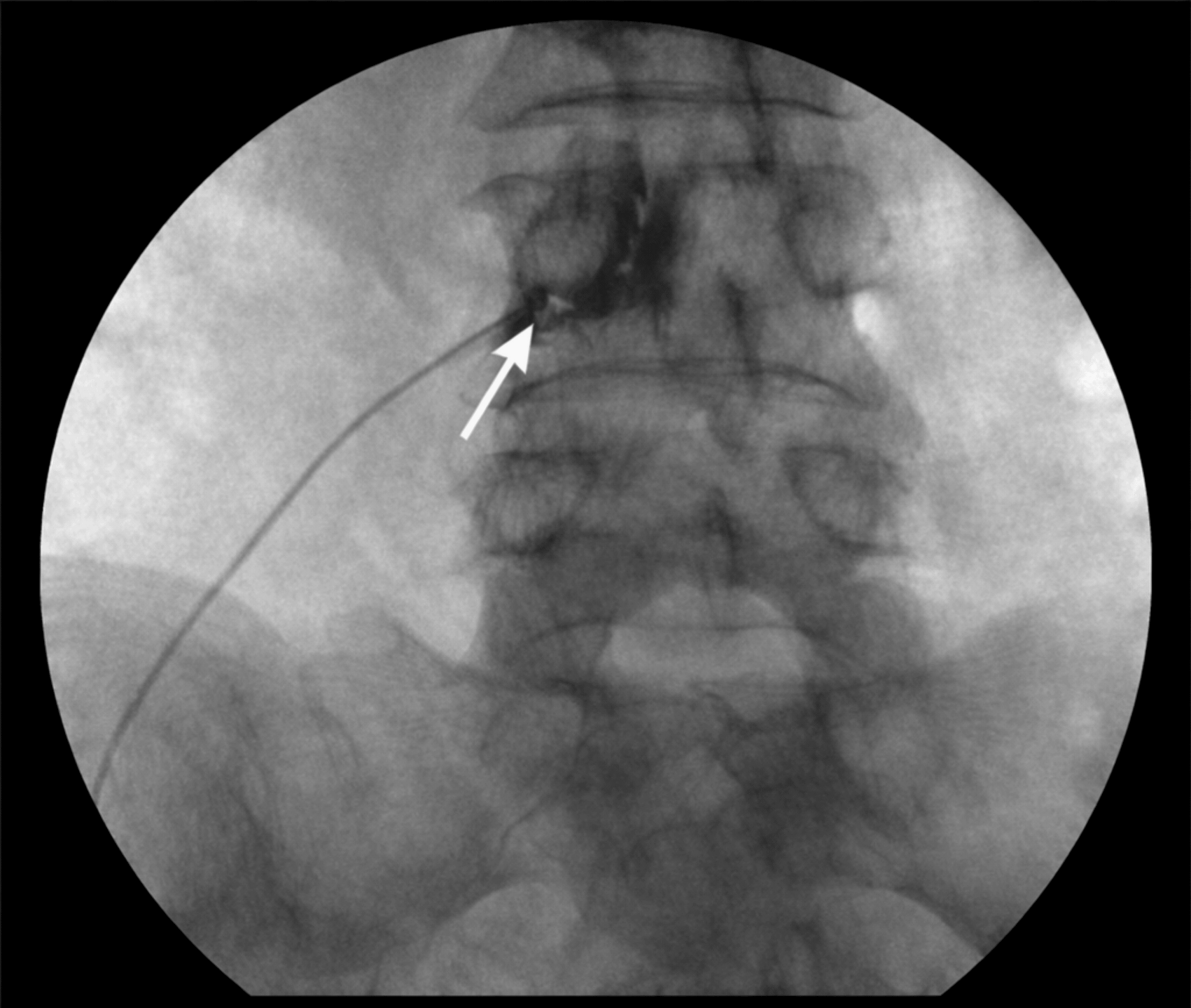
Anteroposterior fluoroscopic view of the left L4 dorsal root ganglion targeting An anteroposterior fluoroscopic view showing the final needle position at the left L4 neural foramen. The needle tip (white arrow) lies just inferior to the L4 pedicle, corresponding to the anatomical location of the L4 dorsal root ganglion.

**Figure 2:**
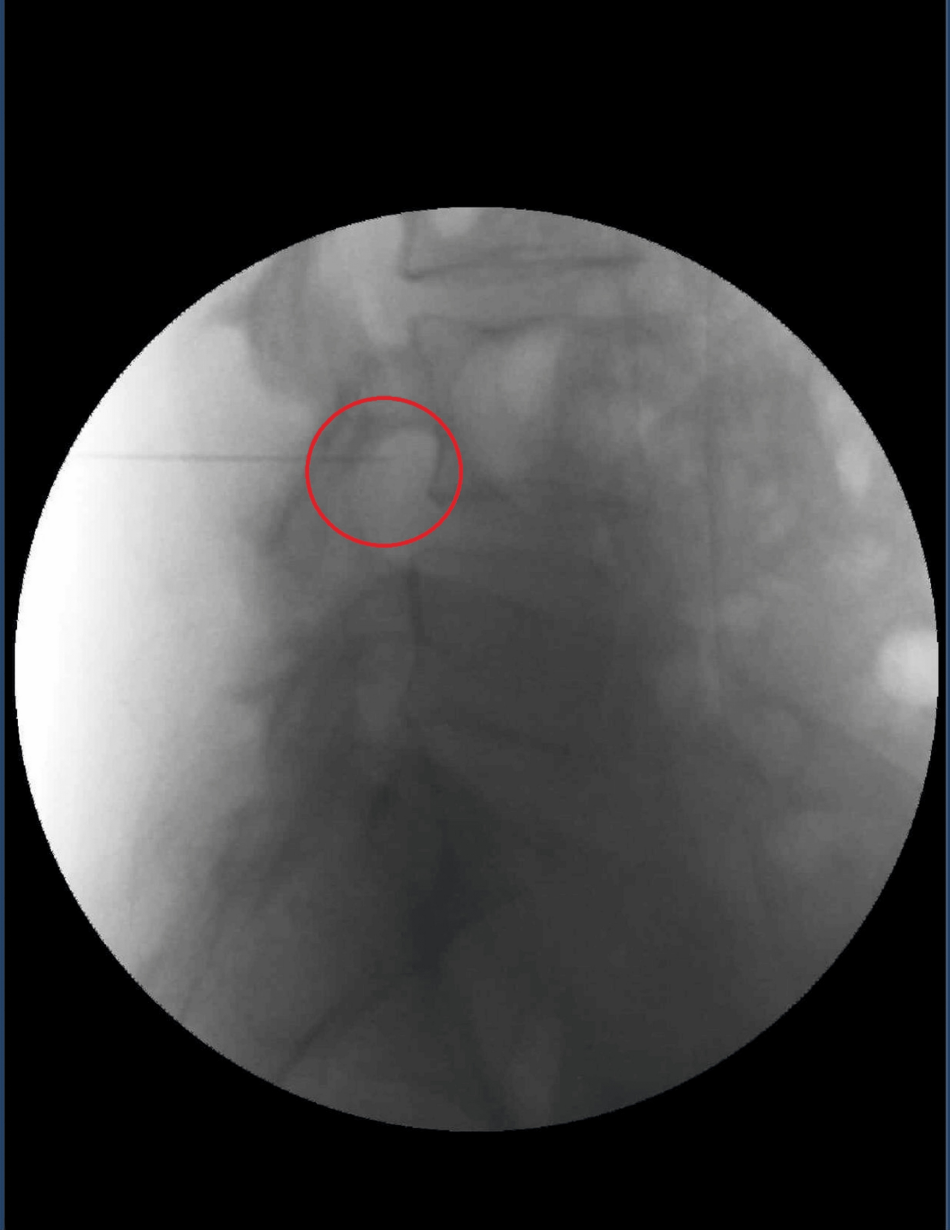
Lateral fluoroscopic view during left L4 dorsal root ganglion pulsed radiofrequency Lateral fluoroscopic view demonstrating needle positioning at the left L4 neural foramen targeting the dorsal root ganglion. The red circle highlights the region of the L4 dorsal root ganglion prior to pulsed radiofrequency application.

In October 2023, an ultrasound-guided therapeutic block of the left saphenous and common peroneal nerves was performed using local anesthetic and steroid. This intervention resulted in improvement of electric shock-like sensations and superficial allodynia but failed to relieve the persistent deep knee pain. Plain radiographs demonstrated satisfactory prosthetic alignment without loosening or signs of infection. Given the persistence of neuropathic symptoms despite multiple peripheral nerve-targeted interventions, a proximal pain generator was suspected. Lumbar radiculopathy was considered based primarily on clinical features, including dermatomal pain distribution and neuropathic characteristics, despite the absence of significant spinal canal stenosis or nerve root compression on lumbar magnetic resonance imaging. The lack of sustained response to peripheral nerve blocks and genicular nerve radiofrequency further supported a non-peripheral pain generator. In December 2024, a fluoroscopy-guided diagnostic left L4 selective nerve root block was performed, resulting in marked pain reduction, improved sleep, and functional recovery lasting approximately two weeks. Based on the positive diagnostic response, a therapeutic left L4 selective nerve root block using local anesthetic and steroid combined with DRG PRF was performed in February 2025, as shown in Figure 1 and Figure 2.

At the five-week follow-up, the patient reported more than 70% pain reduction, with NRS reduced to 1/10, improved sleep, increased mobility, and the ability to resume routine activities. Analgesic medications were discontinued, and physiotherapy was continued as tolerated. Functional outcomes were evaluated using a structured clinical assessment of activities of daily living (ADL). 

Follow-up assessments were conducted at three months, six months, and one year after the intervention. Pain intensity was assessed using the NRS. The patient’s baseline NRS score of 8/10 decreased to 1/10 at the five-week follow-up and remained stable at 1-2/10 at the three-month, six-month, and one-year assessments, corresponding to a sustained pain reduction exceeding 70%.

Functional outcomes were evaluated based on ADL, including standing, walking, sitting tolerance, sleep quality, and independence in routine activities, all of which showed marked improvement compared with baseline. Patient satisfaction was assessed using a self-reported satisfaction index and was rated as very satisfied at all follow-up visits. No procedure-related complications or adverse events were observed throughout the follow-up period.

## Discussion

Chronic pain following TKR affects approximately 10% to 20% of patients and remains a major cause of disability and dissatisfaction despite technically successful surgery [6]. Importantly, a substantial proportion of patients with persistent pain demonstrate no identifiable mechanical or infectious cause on imaging, suggesting neuropathic and centrally mediated pain mechanisms [11]. Neuropathic pain after TKR is increasingly recognized, with reported prevalence rates between 6% and 13% [7]. Potential mechanisms include peripheral nerve injury, neuroma formation, altered central pain processing, and increased excitability of the DRG. Patients with neuropathic features consistently report worse functional outcomes and higher pain scores following TKR [12]. Interventional management strategies described in the literature predominantly focus on peripheral targets, particularly genicular nerve blocks and radiofrequency ablation. Randomized controlled trials have demonstrated that ablation of the superior medial, superior lateral, and inferior medial genicular nerves can provide meaningful pain relief in selected patients [13,14]. Other reported modalities include peripheral nerve stimulation, DRG stimulation, and spinal cord stimulation, particularly in refractory cases [15-17]. Lumbar radiculopathy, particularly involving the L4 nerve root, is an important but frequently overlooked source of referred knee pain due to overlapping dermatomal distributions [3]. Notably, radicular pain may occur even in the absence of radiologically significant compression, a phenomenon attributed to chemical radiculitis and DRG sensitization [18]. This highlights the importance of clinical judgment in the evaluation of chronic pain syndromes, as imaging findings may not always correlate with symptom severity. In the present case, the dramatic yet temporary response to a diagnostic L4 selective nerve root block confirmed a radicular contribution to the patient’s persistent postoperative knee pain. PRF of the DRG offers a minimally invasive, non-destructive neuromodulatory approach. Unlike continuous radiofrequency ablation, PRF maintains tissue temperatures below 42°C, avoiding neural injury [19]. Proposed mechanisms include modulation of synaptic transmission, suppression of ectopic neuronal firing, and reduction of inflammatory mediators within the DRG [20]. Systematic reviews support the efficacy and safety of DRG-targeted PRF in the management of lumbosacral radicular pain [21]. Reports specifically addressing DRG-targeted PRF for refractory post-TKR knee pain are extremely limited. To our knowledge, published data describing L4 DRG pulsed radiofrequency as a treatment for chronic post-TKR pain are scarce, highlighting the novelty and clinical relevance of this case.

## Conclusions

Chronic pain following TKR is a complex condition involving both peripheral and central mechanisms. This case highlights the importance of considering lumbar radiculopathy, particularly L4 involvement, in patients with neuropathic knee pain refractory to conventional and peripheral interventions. Diagnostic selective nerve root blocks can identify a radicular pain component, and DRG PRF can provide substantial and sustained relief. Incorporating DRG-targeted strategies into individualized treatment plans may significantly improve outcomes in carefully selected patients with persistent post-TKR pain.

This report describes a single patient, limiting generalizability. Imaging did not demonstrate overt radiculopathy, emphasizing the value of functional diagnostic tools such as selective nerve root blocks. Future prospective studies are needed to better define patient selection criteria, long-term efficacy, and the role of DRG-targeted interventions within standardized post-TKR pain management algorithms.
